# Does surface priming increase the bond strength of orthodontic brackets? An experimental study

**DOI:** 10.1002/cre2.888

**Published:** 2024-05-07

**Authors:** Sara M. Al Taweel, Spiros Zinelis, Arif Sofyan, Youssef S. Al Jabbari

**Affiliations:** ^1^ Department of Prosthetic Dental Sciences, College of Dentistry King Saud University Riyadh Saudi Arabia; ^2^ Dental Biomaterials Research and Development Chair, College of Dentistry King Saud University Riyadh Saudi Arabia; ^3^ Department of Biomaterials, School of Dentistry National and Kapodistrian University of Athens Athens Greece

**Keywords:** metal primer II, provisional materials, orthodontic bracket, shear bond strength

## Abstract

**Objective:**

To evaluate the effects of metal primer II (MP II) on the shear bond strength (SBS) of orthodontic brackets bonded to teeth and bis‐acryl composite provisional material (Bis‐Acryl).

**Material and Methods:**

Twenty extracted human premolars specimens and 20 premolar shaped Bis‐Acryl specimens were obtained and randomly divided into two surface groups. The first group consisted of human premolars (T) bonded to brackets in the conventional way while in the second (T‐MP) MP II was applied on the bracket base before bonding. Similarly, one group of provisional material (PM) was prepared according to conventional treatment and another with the application of MP‐II metal bonder (PM‐MP). In all cases Ortho‐brackets (Victory Series, 3 M) were bonded employing Transbond XT resin cement. Then the brackets were debonded under shear and the results were statistically analyzed by two‐way analysis of variance and Holm Sidak at *α* = .05. The debonded surfaces of all specimens were examined by light microscopy and the Adhesive Remnant Index (ARI) was recorded.

**Results:**

The SBS results exhibited significant differences er (*p* < .001). For both the T and TM the application of MP‐II increased the SBS compared to respective control groups (*p* < .001). The T‐C group was found inferior compared to PM‐C (*p* < .001) and the same is true for the comparison between T‐MP and PM‐MP (*p* < .001). ARI indexes demonstrated that the tooth groups were characterized by a predominantly adhesive failure at the resin‐dentin interface. In contrast, the control group for provisional crowns (PM‐C) showed a predominantly cohesive failure mode, which moved to predominantly adhesive after the application of MP II.

**Conclusion:**

The application of MP II enhances the SBS on both, human enamel and provisional crown materials.

## 1 INTRODUCTION

The bonding of orthodontic brackets to tooth surfaces is an essential step in orthodontic treatment. However, occasionally the brackets must be bonded to provisional crown materials, since, in many cases, orthodontic treatment is carried out in conjunction with ongoing dental restorative procedures especially for the elder population. Generally, the method of attachment must allow for the distribution of orthodontic forces, and the bond must be sufficiently strong and durable to withstand mastication loads over the entire period of orthodontic treatment. To avoid excess time and cost for the patient and the dentist, bracket debonding should be eliminated during clinical function (Guess et al., 1988; Ireland & Sherriff, 1994; Knoll et al., 1986) while the brackets should be easy to remove at the end of the treatment and result in minimal hard‐ and soft‐tissue damage (Knox et al., 2000). However recent studies provided an average of approximately 6% failure rate of metallic brackets under clinical conditions (Rai, 2015; Romano et al., 2012) while the posterior region was reported as more vulnerable to bracket debonding compare to the anterior (Romano et al., 2012). It is noteworthy to mention that a recent systematic review indicated that the incidence of bracket debonding during orthodontic therapy ranged from 0.6% to 28.3%, characterizing this incidence as very high (Almosa & Zafar, 2018).

Orthodontic brackets are routinely bonded to enamel by the acid‐etch technique in conjunction with a composite‐type orthodontic adhesive. When direct‐bonding metal bases are used, the failure site has been identified mostly at the adhesive‐bracket interface or within the adhesive, both in vitro and in vivo (Algera et al., 2008; Kechagia et al., 2015; Pont et al., 2010). For this reason, the adhesive failure of brackets remains a limitation during treatment. Therefore, various surface treatments such as sandblasting (Arici et al., 2006; MacColl et al., 1998; Ozer & Arici, 2005), silicoating (Newman et al., 1995) and self‐etching primer (Elekdag‐Turk et al., 2008) of the metal bracket surface have been developed, which seem to be effective in enhancing bond strength at the bracket‐adhesive interface.

Today, several adhesive primers containing different functional monomers for bonding resin to metal are commercially available for direct in‐mouth repair of damaged veneers and laboratory applications. Metal primer II (MP II) contains the functional monomer thiophosphoric methacrylate, which promotes bonding due to copolymerization with acrylic resins and composites, as well as chemical reaction to metallic surfaces of all precious and base dental alloys (Freitas & Francisconi, 2004; Imai et al., 2014; Matsumura et al., 2000; Silikas et al., 2007). MP II is indicated for a vast spectrum of repairs for fixed and removable prosthodontic restorations, and current research has reported that it increases the bond strength of acrylic resin to precious and base alloys (Kim et al., 2002; Matsumura et al., 1999; Radhi et al., 2008; Sarafianou et al., 2008).

However, the use of MP II is also indicated for increasing the bonding of metal orthodontic brackets (America, 2008) to adhesive resin, a claim which has not yet been tested experimentally. It is worth noting that orthodontic brackets are made of completely different alloys compared with Au, Pd, Ni–Cr, Co–Cr, and Ti dental alloys, since they are made mainly with austenitic or martensitic stainless steel alloys (Darabara et al., 2007; Eliades et al., 2003, 2008). Therefore, the aim of this study was to characterize the effect of MP II on the bond strengths of orthodontic brackets to enamel and provisional crown materials. The null hypothesis was that no differences will identified between control groups and groups treated with MP II in both human enamel and provisional crown materials.

## 2 MATERIALS AND METHODS

### 2.1 Materials tested

All methods were carried out in accordance with relevant guidelines and regulations. The study was initiated after securing Internal Review Board (IRB). The samples in this study were prepared with human mandibular premolars, stainless steel lower premolar brackets (Victory Series, 3 M Unitek) and a bis‐acryl composite resin provisional material, Protemp 4 Garant (3 M ESPE). An adhesive resin (Transbond XT Light Cure Adhesive Paste; 3 M Unitek) was used to bond orthodontic brackets to tested substrates. Given that this is the first time that metal primer is used for bracket bonding there are no previous studies for a proper sample size calculation. Therefore, the number of 10 specimens per group was adopted which is a common number for decades of published papers dealing with the shear bond strength of orthodontic brackets.

### 2.2 Preparation of tooth specimens

Twenty human mandibular premolars free of caries and restorations were used in this study. The labial and lingual surfaces were cleaned with fluoride‐free pumice in a rubber cup, rinsed with tap water, and dried. Each tooth was mounted in chemically cured dental acrylic (Palavit G, Heraeus Kulzer) in plastic cylinders (23‐mm diameter) to allow for standardized and secure placement during testing.

#### 2.2.1 Conventional method

The specimens were etched with 37% phosphoric acid for 30 s, washed for 20 s, and dried with oil‐free compressed air for 20 s, after which a thin uniform coat of primer (Transbond XT Primer; 3 M Unitek) was applied. The adhesive resin (Transbond XT Light Cure Adhesive Paste; 3 M Unitek) was placed onto the bracket base, and the bracket was positioned on the specimen surface. Excess adhesive resin was removed with an explorer. Adhesive resin was polymerized using curing light (Ortholux LED; 3 M Unitek) at approximately 1000 Mw/cm^2^ for a total of 10 s from two directions (mesial and distal).

#### 2.2.2 Treatment with MP II

The bracket base was sandblasted with 50‐µm airborne alumina grains in a Microetcher ER (Danville Engineering Inc.) at 0.5 MPa propulsion pressure for 15 s over a distance of 10 mm. A thin layer of MP II (GC America Inc.) was then applied to the bracket base by means of a clean brush and allowed to dry according to the manufacturer's instructions. The brackets were then bonded following the procedure as described above.

### 2.3 Preparation of provisional crown specimens

Twenty specimens of the bis‐acryl composite resin provisional material Protemp 4 Garant (3 M ESPE) were fabricated in the shape of premolars by means of a rubber mold of a natural premolar tooth. After the mold had set, the bis‐acryl provisional material was injected into the mold and allowed to set. After setting, the specimens were removed from the mold and embedded in chemically cured dental acrylic. Then the specimens were ground with water coolant SiC papers up to 2000grit in a grinding/polishing machine (Polo250/3, Jean Wirtz) and polished with a polishing paste of (Buehler, Micropolish II, Lake Bluff, Ill), 1 and 0.05 μm particle size. All surfaces were sanblasted with airborne alumina particles (50 μm) for 10 s from a distance of 10 mm employing 0.55 MPa propulsion pressure (Microetcher ER, Danville Engineering Inc). The specimens were then equally divided between two groups (PM‐C and PM‐MP) and brackets were bonded by the two different bonding procedures as described previously for tooth specimens.

### 2.4 Bracket debonding procedure

The shear bond test was performed in a universal testing machine (Instron 8500 R; High Wycombe) at a crosshead speed of 1 mm/min and the shear bond strength (SBS) was calculated in Mpa by dividing the debonding load by the bracket surface area. The debonded surfaces of all specimens were examined by light microscopy at 25× magnification, and the adhesive remnant index (ARI) was recorded based on the scale 0 (totally adhesive), 1 (predominantly adhesive, meaning that less than half the adhesive resin remained on the substrate surface), 2 (predominantly cohesive, meaning that more than half the adhesive resin remained on the substrate surface), and 3 (all resin adhesive left on the enamel).

### 2.5 Statistical analysis

The bond strength data were first analyzed for the presence of outliers employing the estimation of Q25% and Q75% quartiles and interquartile range (ΙQR = Q75%–Q25%). Then the data above Q75% + 1.5 × IQR or below Q25% − 1.5 × IQR were considered as outlier and excluded from the group. Finally, the groups were tested for normality and homscedacity by Shapiro–Wilk and equal variance tests, respectively. The SBS data were analyzed by two‐way analysis of variance (ANOVA), with material type (teeth or provisional material) and the application of primer as discriminating variables (*α* = .05). The significant differences among different groups were determined by the Holm–Sidak pairwise multiple comparison test (*α* = .05). All statistical tests were carried out employing SigmaStat software (SigmaPlot v.12.5, Systat Software Inc).

## 3 RESULTS

Only two outliers were detected in the PM‐MP group and were excluded. The SBS data of all groups were found to comply with normality test (*p* = .992) and equal variance test (0.064). SBS The results of ANOVA showed that there was no interaction between materials and primer (*p* = .314) implying that effect of materials (tooth or provisional material) does not depend on the presence or absence of metal primer.

**Figure 1:**
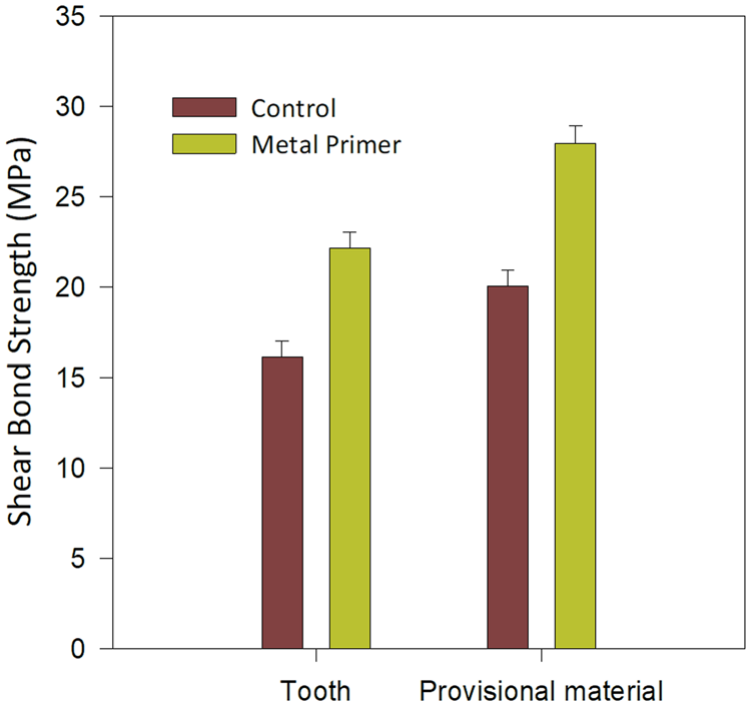
Mean bond strength and standard deviations of all groups included in the study. Only the positive standard deviation is appeared for the sake of clarity. All groups showed statistical significance differences (*p* < .001) in pair comparison between the control and use of MP‐II and between different substrates (teeth vs. provisional material).

The results of shear bond strength (SBS) are presented in Figure 1. (mean values ± standard deviation T‐C: 16.1 ± 1.3, T‐MP: 22.2 ± 2.7, PM‐C: 20.1 ± 3.0 and PM‐MP: 28.0 ± 3.8). All groups showed significant differences among each other with *p* < .001. In both cases the application of MP‐II increased the SBS compared to the respective control groups (*p* < .001). The T‐C groups was found inferior compared to T‐MP (*p* < .001) and the same is true for the comparison between T‐MP and PM‐MP (*p* < .001).

**Figure 2:**
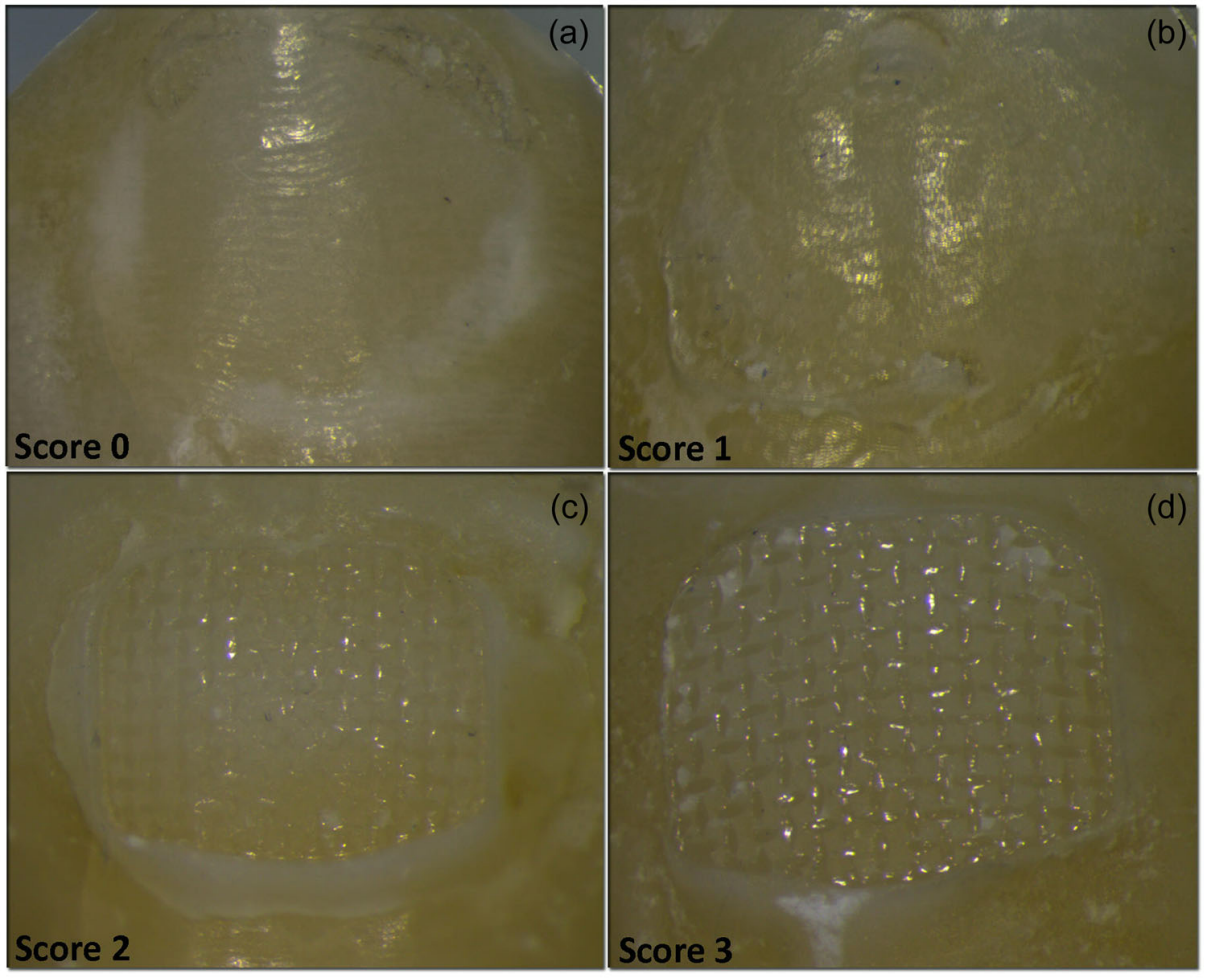
Representative images from the enamel surface after bracket debonding showing the four scores of Adhesive Remnant Index (ARI) (a) no adhesive left on the enamel (score 0); (b) less than half of the adhesive left on the enamel (score 1); (c) more than half of the adhesive left on enamel (score 2); and (d) all adhesive left on the enamel (score 3).

**Figure 3:**
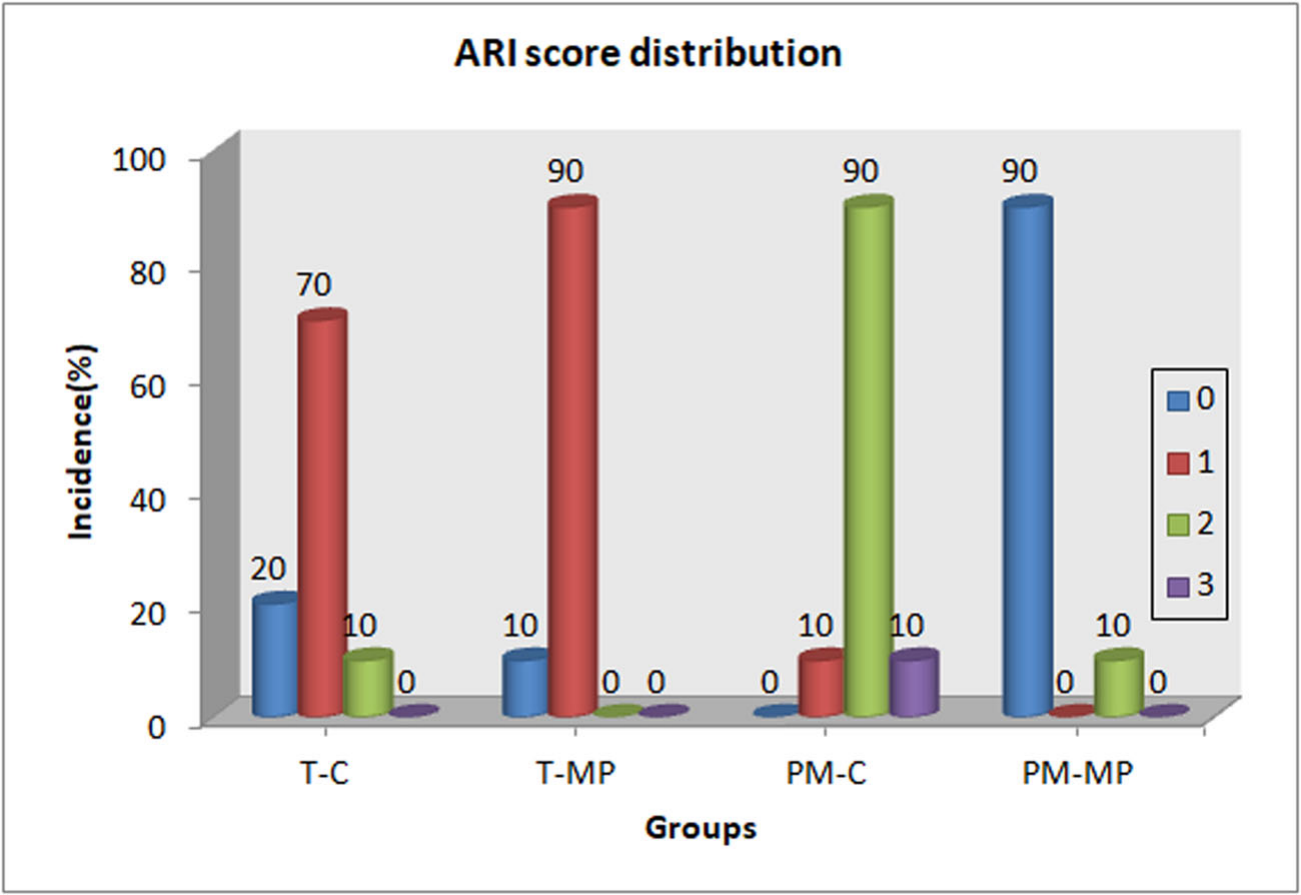
ARI score distribution for groups with brackets bonded to human enamel and provisional crown material.

Figure 2 illustrates representative images from the ARI scale while the distribution of ARI scores for all groups tested is presented in Figure 3. The tooth groups were characterized by a predominantly adhesive failure at resin–dentin interface. In contrast, the control group for provisional crowns (PM‐C) showed a predominantly cohesive failure mode, which moved to predominantly adhesive after the application of MP II.

## 4 DISCUSSION

The application of MP II increased the SBS in both enamel and provisional crown materials, and thus the null hypothesis must be rejected.

The values of SBS for the T‐C group were found to be in accordance with previously reported data (14.99 ± 2.64 MPa) in which the same adhesive resin (Transbond XT) and bracket type (Victory) (Flores et al., 2015) were tested. Similar SBS values were identified in the literature for the same adhesive resin but different orthodontic brackets (15.7 ± 6.7 [Ibarra et al., 2023], 11.06 ± 3.63 [Fernandes et al., 2015], 13.51 ± 1.68 [Kim et al., 2015], and 16.59 ± 6.82 MPa [Blocher et al., 2015]). Although the use of ARI has been criticized for its inherent limitations to characterize the fracture mode (Al Jabbari et al., 2014), it was used in this study to provide qualitative information on the mechanically inferior region in this two interface system (dentin or provisional material‐adhesive resin and adhesive resin bracket) and also for comparison with published data. The ARI score distribution was dominated by “score 1,” a finding in agreement with previous research in which the same adhesive resin and bracket were used (Flores et al., 2015) and in which different brackets were tested (Fernandes et al., 2015). The highest SBS of the MP II surface treatment group might be due to the result of the combination of micromechanical retention, achieved by surface‐roughening by air abrasion with alumina particles, and chemical retention, achieved by the MP II, which provided adhesive bridges between the metal surface and composite resin. Air abrasion with 50 μm aluminum oxide grains on the surface of stainless steel favors micromechanical bonding to resin, promoting the formation of surface irregularities in the metal. The application of MP II to the bracket surface might partially fill these irregularities, forming co‐polymerization with the resin, an approach that would explain the high SBS of the MP II group. It is generally proposed that “Chemical bonding of metal to composite resins” is established when primers that contain functional monomers which create chemical bonding between the resin restorative material and the substrate (Freitas & Francisconi, 2004; Kim et al., 2015; Silveira de Araujo et al., 2006). In general, it has been reported that the use of MP II enhances the bond strength between different types of metal alloys and composite resin materials (Sarafianou et al., 2008; Yanagida et al., 2001; Yoshida et al., 2001).

In this study, the bis‐acryl composite resin provisional material was used because it has been reported to have the highest shear bond strength value when compared with that of other provisional materials (Al Jabbari et al., 2014; Maryanchik et al., 2010; Rambhia et al., 2009). Similarly to the T‐C group, the SBS of PM was found to be close to previously published data (Al Jabbari et al., 2014; Chay et al., 2007). PM groups showed significantly higher SBS to human enamel groups, a finding which should be associated with a completely different adhesive mechanism of orthodontic resin to biological tissues and a Bis‐acrylic composite resin. Given that the surface roughening and metal primer is applied at the bracket resin interface the testing of SBS with provisional material may be considered irrelevant. However this is not true as the extent and distribution of developed stresses during shear loading is dependent on the mechanical properties of the jointed materials (Braga et al., 2010; DeHoff et al., 1995). Interestingly the ARI shifted from predominantly 2 (cohesive fracture of adhesive resin) to adhesive fracture at the resin provisional material interface. This shifting may be explained by the enhanced strength of adhesive resin‐bracket joint provided by MP II, which further protects the interface from crack nucleation and propagation leaving the adjacent region (adhesive resin–dentin or provisional material interface) more vulnerable to fracture.

According to the manufacturer's instruction the metallic surface must be sandblasted with alumina grains before the application of MP‐II and thus the increase in SBS should be associated with the synergistic action of both factors which are micromechanical retention and chemical bonding. Therefore, further research should be done with groups omitting sandblasting in the case that the discrimination of the effect of each contributing factor is needed. In contrast to fixed and partial dentures, whose surfaces are well‐polished before the application of MP II, and thus require roughening to improve wettability and micromechanical bonding, the mesh surfaces of orthodontic brackets provide macro‐irregularities (Kechagia et al., 2015; Zinelis et al., 2005) to improve mechanical retention with adhesive resin, making the contribution of microirregularities questionable. The inability to discriminate the effect of two aforementioned factors should be considered as a limitation of the current study, but we have to follow the manufacturer's instruction in this first approach.

The value of the finding of this study from a clinical standpoint of view is that orthodontist may consider applying a metal primer MP II in anterior areas where brackets are more likely to fail during orthodontic treatment (Romano et al., 2012) despite the additional chairtime required. However, the beneficial effect of MP‐II requires data based on further experimental testing employing artificial aging regimes (i.e., water thermocycling) and clinical evidence and thus its failure rate should be further investigated with randomized clinical trials before this technique can be proposed as a valuable addition in orthodontic treatment.

## 5 CONCLUSIONS

MP II has a beneficial effect on both human teeth and provisional material.

## AUTHOR CONTRIBUTIONS

All authors equally contributed to all parts of this research.

## CONFLICT OF INTEREST STATEMENT

The authors declare no conflict of interest.

## ETHICS STATEMENT

All methods were carried out in accordance with relevant guidelines and regulations. The study was initiated after securing the Internal Review Board (IRB).

## Data Availability

All data generated and/or analyzed during this study are included in this published article.
